# Online prediction of others’ actions: the contribution of the target object, action context and movement kinematics

**DOI:** 10.1007/s00426-012-0423-2

**Published:** 2012-03-08

**Authors:** Janny C. Stapel, Sabine Hunnius, Harold Bekkering

**Affiliations:** Donders Institute for Brain, Cognition and Behaviour, Radboud University Nijmegen, P.O. Box 9104, 6500 HE Nijmegen, The Netherlands

## Abstract

Previous research investigated the contributions of target objects, situational context and movement kinematics to action prediction separately. The current study addresses how these three factors combine in the prediction of observed actions. Participants observed an actor whose movements were constrained by the situational context or not, and object-directed or not. After several steps, participants had to indicate how the action would continue. Experiment 1 shows that predictions were most accurate when the action was constrained and object-directed. Experiments 2A and 2B investigated whether these predictions relied more on the presence of a target object or cues in the actor’s movement kinematics. The target object was artificially moved to another location or occluded. Results suggest a crucial role for kinematics. In sum, observers predict actions based on target objects and situational constraints, and they exploit subtle movement cues of the observed actor rather than the direct visual information about target objects and context.

## Introduction

From very early in life, humans do not only just passively observe other people’s actions but also predict their action goals while watching the actions unfold (see e.g., Falck-Ytter, Gredebäck, & von Hofsten, 2006; Stapel, Hunnius, van Elk, & Bekkering, 2010; Hunnius & Bekkering, 2010). Predicting others’ actions is essential in understanding the other (Blakemore & Decety, 2001), and allows us to smoothly interact with each other (Sebanz, Bekkering, & Knoblich, 2006). When observing actions, there are several sources of information which can form the basis of these predictions. Goal objects, together with situational constraints, the actor’s movement kinematics, and the action path itself, together make up an action (Cuijpers, van Schie, Koppen, Erlhagen, & Bekkering, 2006). Although it is clear that all these factors might affect action prediction, they have to date never been examined together in one empirical study. Especially the role of movement kinematics in combination with other (competing or confirming) information is unclear. That is, on the one hand, it is obvious that there is a “tight coupling between kinematics and goals” (Grafton & Hamilton, 2007, p.609), on the other hand, both behavioral (Bach, Knoblich, Gunter, Friederici, & Prinz, 2005; van Elk, van Schie, & Bekkering, 2008) and neuroimaging data (Grafton & Hamilton, 2007) suggest goals to be more prominent than movement kinematics in action perception. The current study is the first to investigate the role of goal objects, environmental constraints, and movement kinematics for predictions about the action path of an observed actor.

How people come to predict others’ actions has been studied with different paradigms, all contributing pieces to the puzzle of which sources in the visual domain may be used for these action predictions. In general, empirical studies mainly have explored how these sources contribute to action prediction in isolation. Theoretical models, on the other hand, have to some extent focused on combined sources for action prediction, as they all incorporate contextual constraints and goals as major factors. According to Gergely & Csibra (2003) and Baker, Saxe, & Tenenbaum (2009), humans predict actions of intentional agents by assuming that they take the most efficient path to get to a certain goal. The presence and position of environmental constraints, such as barriers, determine which path is most efficient for the agent to take. Hence, one can predict the action path based on information about the goal of an action and the action constraints. Some models include movement kinematics as a third factor explaining action prediction, besides goal and action constraint information (see e.g., Cuijpers et al., 2006; Kilner, Friston, & Frith, 2007). According to Kilner et al. (2007), action predictions are generated by the mirror neuron system (MNS), and are based on information from observed movement kinematics (lowest level), goal inferences (highest level), and contextual information (serving as a prior). Taken together, three aspects are mentioned in the literature which can underlie action predictions, namely information about goals, context and movement kinematics.

The contribution of all three factors in isolation to action perception is indicated by several empirical studies. First of all, contextual information can help in assessing and predicting an action goal. The same hand posture can be interpreted as having the action goal “to clean up” or “to drink”, based on a different context in which the hand is displayed, and the inferior frontal gyrus (which is suggested to be part of the human MNS, see also Rizzolatti & Craighero, 2004) responds differently in these two cases (Iacoboni et al., 2005). The presence or absence of contextual constraints, such as obstacles, can lead to different predictions about an action path. For instance, infants’ expectations seem violated when an agent makes a detour which is no longer ‘needed’, because, an obstacle is removed from the scene (Gergely, Nádasdy, Csibra, & Bíro, 1995; but see: Paulus, Hunnius, van Wijngaarden, Vrins, van Rooij, & Bekkering, 2011b). Adults also seem to take action constraints into account when making predictions about which goal location an agent is heading for (Baker et al., 2009).

Second, goal objects and locations have been shown to have a considerable impact on action prediction. Observing objects which can function as an action goal leads to predictions about what action will follow (see e.g., Tucker & Ellis, 2004). Furthermore, when viewing objects and associated actions, observers generate predictions about goal locations (van Elk, van Schie, & Bekkering, 2009; Hunnius & Bekkering, 2010). Moreover, results from neuroimaging studies illustrate that observed object-directed actions are processed differently in the brain than intransitive actions. For instance, observation of object-directed actions leads to stronger effects in cortical motor areas than non-object-directed actions (Muthukumaraswamy, Johnson, & McNair, 2004; Buccino et al., 2001; Caspers, Zilles, Laird, & Eickhoff, 2010). Furthermore, observation (and simulation) of object-directed actions is tends to activate different regions in the parietal lobe compared to intransitive actions (Jeannerod, 1994; Lui et al., 2008; Creem-Regehr & Lee, 2005).

Third, action kinematics can be used in understanding and predicting the observed actions. For instance, participants can judge based on body movements of actors whether the weight they lift corresponds to the weight they expect (Grèzes, Frith, & Passingham, 2004a), and whether lifting a certain weight was pretended or real (Grèzes, Frith, & Passingham, 2004b). Furthermore, the intention underlying a grasping movement (to cooperate, compete or to perform an individual action) can be accurately predicted when the start of this movement is observed (Sartori, Becchio, & Castiello, 2011). Even when the action seems to have no target object, accurate predictions about an observed action can be made on-line when watching movement kinematics (Graf, Reitzner, Corves, Casile, Giese, & Prinz, 2007). Predicting the flow of these observed movement kinematics is easier when an observed point-light figure displays human kinematics compared to less complex non-human kinematics, which suggests that the motor system maps observed actions to come to predictions of the observed action (Stadler, Springer, Parkinson, & Prinz, 2012). In addition, in real life tasks, such as in joint action settings, people not only predict the goal of another person’s action but also the action kinematics necessary to achieve this goal. This is illustrated by the finding that people adjust their behavior such that beginning state comfort is attained for an interaction partner (Gonzalez, Studenka, Glazebrook, & Lyons, 2011).

In sum, previous research demonstrates that contextual constraints, goal objects as well as action kinematics can be used for action prediction. However, how these three aspects together contribute to action predictions of human actions remains unclear. Especially, the role of movement kinematics opposed to more abstract object and context information needs further investigation. Theoretically, action predictions could be solely based on the combination of situational constraints and target objects. However, when simulating an observed action, movement kinematics may also play a role in the prediction how an observed action will unfold. The current research question, thus, was two-fold. Do people take situational constraints and target objects into account when predicting how an observed ongoing action will unfold? And if so, do they at least partially rely on the movement kinematics in making their predictions? Experiment 1 was designed to answer the first question. There, predictions had to be made about the subsequent movement of an observed actor, while the action was object-directed or not, and was constrained by the context or not. Experiment 2A and 2B allowed us to examine whether predictions were made purely on the information about the goal object in combination with the context of the action, or whether the predictions were based on the actor’s movement kinematics. The previous work in the area of action observation suggests that action representations are hierarchically organized (Grafton & Hamilton, 2007), such that incongruent information from means is less detrimental than incongruent goal information when processing observed actions (van Elk et al., 2008). In similar fashion, we provided participants in Experiment 2A with movement kinematics which were incongruent with the goal-object and action context. Different theories would generate opposing hypotheses for this conflict in provided information. If action predictions are mainly based on goal-objects and situational constraints, prediction accuracy may show a similar pattern as in Experiment 1. On the other hand, if humans make use of all three sources of information (goal-object, action context, and kinematics) for their action predictions, conflicting information may lead to reduced differences between the conditions. However, if kinematics are driving action prediction, the pattern in the prediction accuracy data of Experiment 1 might be reversed. In Experiment 2B, information about the goal object was no longer available to the participant. If action predictions are mainly based on goal-objects and action context, one would expect to find a main effect of action context, and no effect of object-directedness. Alternatively, when movement kinematics can be used as a basis for action prediction, a more elaborate pattern of accuracy data may be obtained.

## Experiment 1

### Method

#### Participants

Eighteen participants (3 males) with a mean age of 22 years (SD = 4 years) were tested. They gave written informed consent to participate and either chose to receive five euros in vouchers for participation or credit points. All were right-handed students recruited at the Radboud University in Nijmegen.

#### Design

The study was an action-observation setting, in which a two by two within-subjects design was applied. Participants viewed videos of an actor walking a few steps and then crawling. In half of the cases the action was object-directed, in the other half it was not-object-directed (Target object vs. No target object). As a second manipulation, the action context was manipulated such that crawling took place either underneath the table or beside the table (Underneath table vs. Beside table). Halfway the second step of the actor, the video was paused and participants had to judge whether the actor would take another step walking or change to crawling. In 50% of the cases, the correct response would be that the observed actor would start crawling. Responses were given by pressing one of the two response-buttons with the left or right index finger. Between subjects, the response buttons were counterbalanced between left and right hand. Accuracy rate of the responses (correct/incorrect) and the *d* prime (*d’*) of this accuracy rate were the dependent variables.

#### Materials

**Fig. 1:**
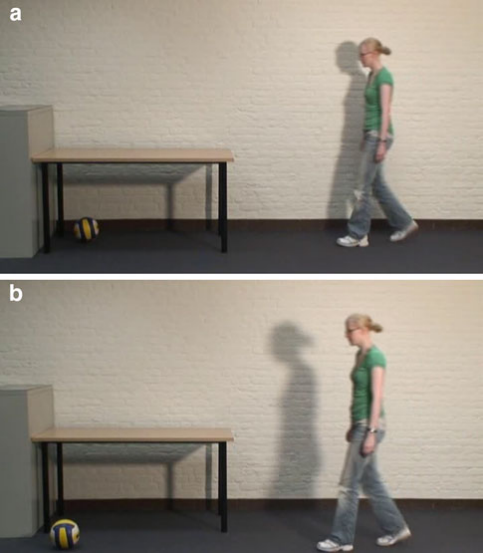
**a** Example frame in which actor will start crawling. **b** Example frame in which actor will continue another step walking

Stimuli were videos which displayed three different female actors standing still for one second, then starting to move with two or three steps walking and then crawling in the same direction, and ending with a still posture of approximately one second. Average stimulus duration was 5.7 s (SD = 0.4 s). Stimulus movies were presented with a frame rate 25 frames per second, were displayed against a black background and were 408 pixels high and 720 pixels wide. In all videos, a table and a volley ball were present. The ball lay either on the floor (Target object condition) or on the table (No target object condition). As participants received the information that the actor would first walk and then crawl, it was clear from the start of the experiment that the action was not object-directed if the ball lay on the table. The table stood in front of a white wall. The actor either moved close to the wall (see Fig. 1a), or a few steps more in front (see Fig. 1b), which made clear from the start of the movie whether the actor would crawl underneath the table (close to the wall) or beside the table (a bit more in front). When video-taping the actions, stimuli were recorded mixing the order of conditions constantly, such that differences in the movements of the actor are not a consequence of having repeated the exact same action in the same context repeatedly. Actors were trained in making stimulus material, and were instructed to act as similar as possible in all their actions. To ensure the similarity between the stimuli, actors were shown example videos before and in-between taping sessions, and were asked to pay special attention to their walking pace, how to end the action in a natural fashion, and the shift from walking to crawling. For each condition, ten different stimuli were used. The stimuli displayed three different actors. However, one of the actors moved in a different way than the other two. That is, she had the tendency to not walk upright, and she moved both her hands before her body when starting to crawl. Nevertheless, her stimulus movies were included to keep some natural variation in the stimuli, but only in two out of every ten stimuli per condition. Furthermore, the movement direction was varied, and between stimuli, there were little changes in the position of the furniture, starting position of the actor, and position of the ball. This was to ensure that exact timing and position of crawling of the actor could not be inferred from having seen the other stimuli. Stimuli were between conditions matched for stimulus duration, movement duration, position of the table, movement direction (left or right), amount of steps before crawling and the horizontal distance to the ball. For all stimuli, the motion energy for the complete videos as well as for the part of the videos before the pause was calculated. Motion energy can be indicative for differences in movements contained in videos (Bobick, 1997; Schippers, Roebroeck, Renken, Nanetti, & Keysers, 2010)**.** Between subjects ANOVAs were conducted to test for differences in the variability (expressed as the SD of the motion energy in the videos) of motion in the movies, with context and object-directedness as explanatory variables. Both ANOVAs showed a main effect of context [Until the pause: *F*(1,36) = 4.4, *p* = 0.04; Complete videos: *F*(1,36) = 82, *p* < 0.001], with larger SDs in the motion energy for the beside table condition (Until pause: *M* = 2,890,012; Complete videos: *M* = 3,286,818) compared to the Underneath table condition (Until pause: *M* = 2,397,975; Complete videos: *M* = 2,241,175). The motion-energy algorithm applied here is the sum of the squared differences in the color channels of each pixel between frames (cf. Schippers et al., 2010). In the Beside tables conditions the actor moves closer to the camera, and takes up a larger area, and hence more pixels, of the stimulus, which could explain the results of the motion energy ANOVAs. Alternatively, it might be that the actors move in a less-variable manner in the Underneath table condition compared to the Beside table condition.

All conditions consisted of 10 different stimuli which were each repeated four times during the experiment. Stimuli were presented in random order. To slow down unwanted habituation effects, we included 15 catch trials (8% of the trials) in which the action path differed from the usual path (e.g., crawling on the table, walking beside the table).

The experiment was conducted using a custom-made stimulus presentation and data registration program implemented in Presentation 13.1 (Neurobehavioral Systems, CA, USA). A response-button box was used to log the responses of the participants.

#### Procedure

The experiment started with an instruction phase, in which participants learnt that the stimuli would display an actor who would walk and then crawl. Furthermore, the stimuli would be paused after several steps of the actor, followed by a question: “Will the actor now start crawling, or will she take another step walking?” (see Fig. 1a, b for example frames at which the videos were paused). Participants were instructed to respond as fast and accurate as possible. After their response, the rest of the video would be displayed. Participants practiced with two example stimuli and were provided with feedback about the accuracy of their response. During the actual experiment, no explicit feedback was given, although it could be inferred from watching the rest of the stimulus movie. In total, the experiment took about 30 min to complete. After finishing the experiment, participants were thanked and received participation vouchers or credit points.

### Results

For each participant, the accuracy rate per condition was calculated. Furthermore, mean *d*’ per participant per condition was calculated (see Table 1).

**Table 1:** Mean accuracy rates and d’ per condition for Experiment 1, 2A, and 2B

	Exp.1		Exp. 2A		Exp. 2B	
	Accuracy rate	*D* prime	Accuracy rate	*D* prime	Accuracy rate	*D* prime
Underneath table × Target object	73% (8.6)	1.36 (0.64)	57% (12)	0.30 (0.60)	74% (11)	1.48 (0.74)
Underneath table × No target object	55% (7.0)	0.26 (0.38)	70% (11)	1.29 (0.71)	54% (11)	0.15 (0.57)
Beside table × Target object	57% (7.0)	0.24 (0.44)	59% (8)	0.59 (0.60)	56% (5.8)	0.12 (0.37)
Beside table × No target object	56% (7.2)	0.42 (0.49)	55% (10)	0.06 (0.63)	61% (9.0)	0.75 (0.16)

Standard deviations are noted between brackets

A two-by-two repeated measures ANOVA was conducted with Object-directedness and Context as independent factors, and accuracy rate as dependent variable. A main effect of Context [*F*(1,17) = 12.8, *p* = 0.002], a main effect of Object-directedness [*F*(1,17) = 78.8, *p* < 0.001] as well as the interaction between these two factors [*F*(1,17) = 25.2, *p* < 0.001] were found to be significant. Post hoc paired-samples *t*-tests revealed that accuracy rates were significantly higher in the Underneath table conditions (*M* = 64%) than in the Beside table conditions [*M* = 56%, *t*(17) = 3.6, *p* = 0.002]. Furthermore, the Target object conditions yielded a significantly higher prediction accuracy rates (*M* = 65%) than the No target object conditions [*M* = 55%, *t*(17) = 8.9, *p* < 0.001].

**Fig. 2:**
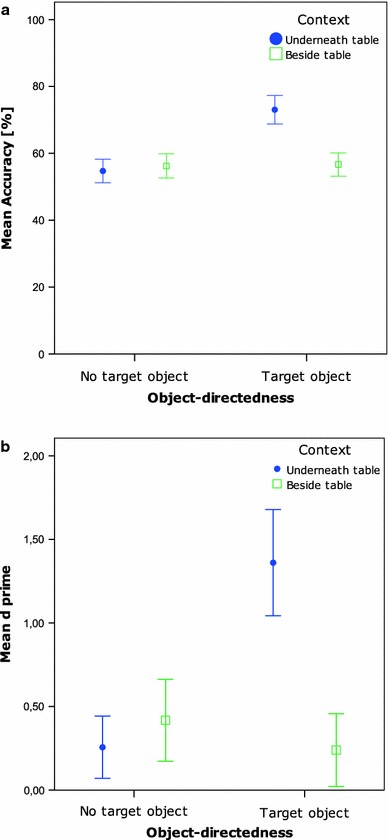
**a** Mean accuracy per condition Exp. 1. *Bars* represent 95% confidence intervals of SE. **b** Mean *d’* per condition Exp. 1. *Bars* represent 95% confidence intervals of SE

As Fig. 2 reveals, the main effects are driven by the interaction effect, which reflects the significantly higher accuracy rates in the condition Underneath table with Target object condition compared to all other conditions [all comparisons with the Underneath table with Target object condition: *t*(17) > 5.8, *p* < 0.001; *t*(17) < 0.8, n.s. for all other comparisons]. The *d’* analysis showed exactly the same pattern of results, with again a significant main effect of Context [*F*(1,17) = 17.1, *p* < 0.001], a significant main effect of Target object [*F*(1,17) = 42.8, *p* < 0.001], and a significant interaction effect of these two factors [*F*(1,17) = 30.5, *p* < 0.001], when applying a two by two repeated measures ANOVA. Post hoc paired-samples *t*-tests investigating the two main effects, show that the *d’*s were higher in the Underneath table (*M* = 0.81) compared to the Beside table conditions [*M* = 0.33; *t*(17) = 4.1, *p* = 0.001], and that the *d’*s of the Target object conditions (*M* = 0.80) were higher than the No target object conditions [M = 0.34; *t*(17) = 6.5, *p* < 0.001]. These two main effects are explained by the interaction effect, with significantly higher *d’*s in the condition Underneath table with Target object compared to all other conditions [all comparisons with the condition Underneath table with Target object: at least *t*(17) > 6.6, *p* < 0.001; *t*(17) < 1.6; n.s. for all other comparisons].

### Conclusion

The data of Experiment 1 show that participants’ predictions of the next move of an actor were more accurate when two things hold: the action was object-directed and contextually constrained compared to the other three combinations. To rule out the possibility that the effect found was a response bias, i.e., participants have a general tendency to react more often with a positive response when the action is object-directed, *d’*s were calculated. *D’* is a measure originating from the sensory detection theory. It is the difference between the *z*-score of the hit rate and the *z*-score of the false alarm rate (Macmillan & Creelman, 1991). The larger this difference, the more sensitive is the measure it reflects. The *d’* analysis yielded the same pattern of results as the accuracy data, showing that the results are not a mere response bias. Furthermore, this suggests that participants become more sensitive in their predictions when there is a target object and the context of the action constrains the actor.

The finding that the accuracy of action predictions in all three other conditions did not differ from each other, suggests that the effect of context and target object are not independent from each other. Apparently, a contextually constrained movement only becomes more predictable if a target object is present. The target object might direct the movements of the actor towards a specific location, which renders the movement more predictable. However, the presence of the target object in itself is apparently not enough to inform the observer about the exact timing of crawling onset of the actor. To predict whether crawling will start immediately after the pause or not, more information seems needed. This information is provided by the contextual constraint. That is, the constraint induces a spatial restriction on the spacing and timing of the transition from walking to crawling, which may increase the predictability of the action. Consequently, the combination of object-directedness and action constraints might lead to more accurate predictions.

From this experiment, it cannot be concluded whether the predictions made are the product of the combination of the goal and context information given by the visual scene, or are possibly derived from the movements of the actor. Therefore as a follow-up, the videos were edited in such a fashion that the target object was placed on a different location in the scene. Consequently, the movement kinematics of Experiment 1 were preserved, but the target information was shifted. Stimulus movies in which there used to be a target object lying on the floor were rendered into movies in which the target object was now lying on the table. The opposite was done with the stimulus movie in which there used to be no action target (as the object had been lying on the table without any function). Here, the object now became the target of the movement (the movie was edited in a way that the object was now lying on the floor). If the most accurate action predictions would still be found in the new Underneath table with Target object condition, this would provide evidence for a role of target information in action prediction. Furthermore, this would show that movement kinematics are neglected by observers when making predictions about an ongoing action, and that situational constraints and target objects are the cornerstones of action prediction (Gergely & Csibra, 2003; Baker et al., 2009). However, if the effect would now be found in the new Underneath table with No target object condition (with the kinematics of the previous Underneath Table with Target object condition), this would support the notion that movement kinematics play a role in action predictions.

## Experiment 2A

### Method

#### Participants

Twenty-eight students (4 males) of age 21 years (SD = 2 years) participated in the study and chose to receive either five euros in vouchers or credit points for participation. All gave written informed consent and were right handed students recruited at the Radboud University in Nijmegen. One participant was excluded from analysis because of computer problems.

#### Design

The same design as in Experiment 1 was applied.

#### Materials

The same stimulus material as in Experiment 1 was used as the basis for Experiment 2. However, all stimulus videos were edited offline beforehand using Adobe Premiere CS 4 (CA, USA). The target object was placed on a different location in the scene. In the Underneath table with Target object movies, the ball was placed on the table, rendering it into an Underneath table with No target stimulus. In the Underneath table with No target condition, the opposite was done: the ball was now placed underneath the table. In a similar fashion, stimuli of condition Beside table with Target object were transformed into Beside table with No target and vice versa (by placing the ball either on the table or on the floor beside the table). Beside the editing of the stimulus materials, no changes were made to the experiment.

#### Procedure

The same procedure was applied as in Experiment 1.

### Results

**Fig. 3:**
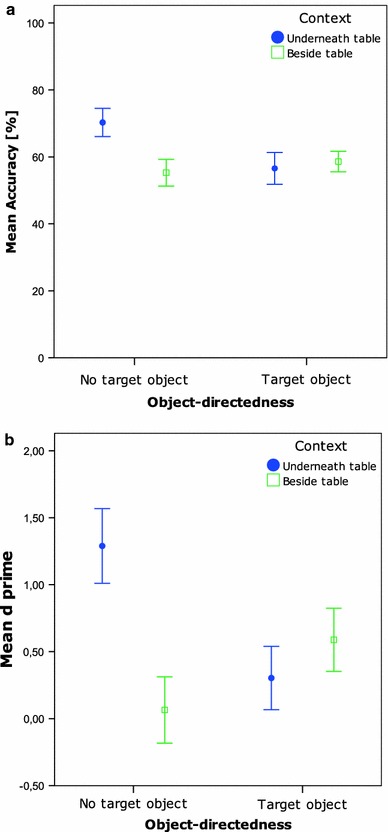
**a** Mean accuracy per condition Exp. 2A. *Bars* represent 95% confidence intervals of SE. **b** Mean *d’* per condition Exp. 2A. *Bars* represent 95% confidence intervals of SE

As in Experiment 1, accuracy rates and *d’*s were calculated per condition per participant (see Table 1). A two (Context) by two (Object-directedness) repeated measures ANOVA revealed that accuracy rates were influenced by both factors [Context: *F*(1,26) = 11.6, *p* = 0.002; Object-directedness: *F*(1,26) = 9.9, *p* = 0.004] and by the interaction of the two [*F*(1,26) = 29.4, *p* < 0.001]. Accuracy rates were significantly higher in the Underneath table conditions (*M* = 63%) compared to the Beside table conditions [*M* = 57%; *t*(26) = −3.1, *p* = 0.004]. The No target object conditions resulted in more accurate action predictions (*M* = 63%) than the Target object conditions [*M* = 58%; *t*(26) = 3.4, *p* = 0.002]. Figure 3a demonstrates that the main effects found were a consequence of the significantly higher accuracy rates when the actor crawled underneath the table with no target compared to the other three conditions [*t*(26) > 5.2, *p* < 0.001 for all comparisons with the Underneath table with No target condition; *t*(26) < 1.8, n.s. for all other comparisons].

An equivalent repeated measures ANOVA was run on the *d’* data and yielded the same pattern of results, namely again two main effects [Context: *F*(1,26) = 18.9, *p* < 0.001; Target object: *F*(1,26) = 5.0, *p* = 0.03] and interaction effect [*F*(1,26) = 51.6, *p* < 0.001]. Post hoc paired-samples *t*-tests, investigating the two main effects, reveal that the *d’*s were higher in the Underneath table (*M* = 0.80) compared to the Beside table conditions [*M* = 0.33; *t*(26) = 4.4, *p* < 0.001], and that the No target object conditions yielded higher *d’*s (*M* = 0.68) than the Target object conditions [*M* = 0.45; *t*(26) = −2.2, *p* = 0.03]. The two main effects and the interaction effect in this ANOVA could be explained by significantly higher *d’*s (*M* = 1.29) for the condition in which the crawling took place underneath the table with no target object compared to the three other conditions [highest other: *M*_Besides_table_x_Target_object_ = 0.59. Comparisons with Underneath table with No target condition were significant: all *t*’s(26) > 4.7, *p* < 0.001]. Other post hoc paired-samples *t*-tests showed that although *d’*s appeared to be higher in the Beside table with Target object condition, this was not a systematic difference [comparison with Beside table with No target: *t*(26) = 4.1, *p* < 0.001; comparison with Underneath table with Target object: *t*(26) = 1.9, n.s.].

### Conclusion

Results of Experiment 2A show a difference in the accuracy of action predictions between conditions. Participants performed significantly better if the action was constrained by the context and not object-directed. The *d’* analysis yielded the same pattern of results, indicating that the effect in the accuracy data is not a mere response bias. Action predictions were more accurate for the stimuli which were in the first experiment object-directed and contextually constrained. Thus, the effect found in Experiment 1 shifted together with the original movement kinematics. This finding suggests that not the target object itself influences the observers’ action predictions, but the movement kinematics of the actor they observed. To further establish this finding, a second manipulation was carried out, in which the target object was not visible in any of the stimuli. This was done by means of an occluder. In this case, the effect could either disappear, indicating that target object information is crucial for action prediction, or it could stay, indicating that movement kinematics help us in making accurate predictions about observed actions.

## Experiment 2B

### Method

#### Participants

In Experiment 2B, 24 participants (four males) took part with a mean age of twenty years (SD = 2 years). All were right-handed students and gave written informed consent for participation. They were recruited at the Radboud University Nijmegen and received afterwards either five euros in vouchers or credit points for participation. For one subject, data could not be recovered because of computer problems.

#### Design

The same design as in Experiment 1 and 2A was applied.

#### Materials

**Fig. 4:**
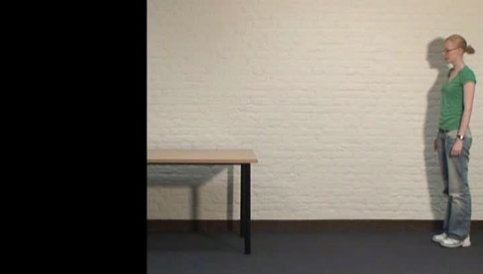
Example of the first frame of a stimulus movie in Experiment 2B

The same stimulus material as in Experiment 1 was used. However, a black occluder was placed over the target object. The dimensions of the occluder were equal for all stimuli, namely 220 pixels wide and 720 pixels high, occluding the right or the left side of the stimulus (depending on the movement direction of the actor), and occluded the target object entirely (see Fig. 4). After the response of the participant, the occluder was removed, showing the complete, original scene. Apart from these changes in the stimulus material, no changes were made to the experiment.

#### Procedure

The same procedure as in Experiment 1 and 2A was applied.

### Results

**Fig. 5:**
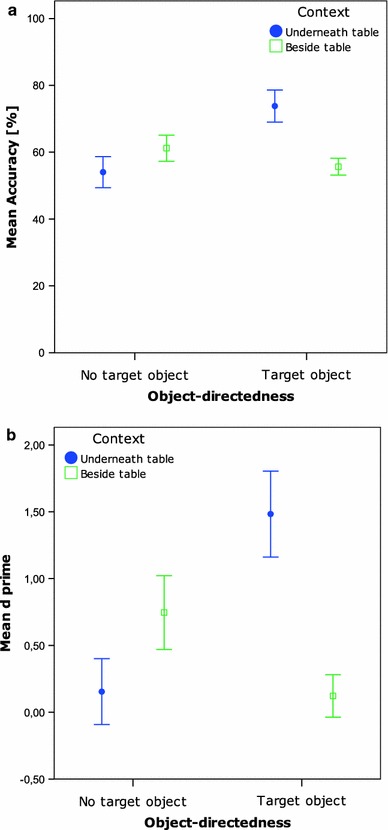
**a** Mean accuracy per condition Exp. 2B. *Bars* represent 95% confidence intervals of SE. **b** Mean *d’* per condition Exp. 2B. *Bars* represent 95% confidence intervals of SE

Comparable to Experiment 1 and 2A, accuracy rates and *d’*s were determined for each participant in each condition (see Table 1). A two by two repeated measures ANOVA showed that Context had a significant impact on the accuracy rates [*F*(1,22) = 7.2, *p* = 0.01], as did the manipulation of the Target object [*F*(1,22) = 20.5, *p* < 0.001], and the interaction between these two factors was also found to be significant [*F*(1,22) = 82.9, *p* < 0.001]. Post hoc paired-samples *t*-test were conducted to verify the direction of the main effects. Action predictions were more accurate in the Underneath table (*M* = 64%) compared to the Beside table conditions [*M* = 58%; *t*(22) = 2.7, *p* = 0.01]. Furthermore, participants responded more accurately when the action had a Target object (*M* = 65%) compared to when there was No target object [*M* = 58%; *t*(22) = 4.5, *p* < 0.001]. Additional post hoc paired-samples *t*-tests were executed to examine the interaction effect. These *t*-tests show that accuracy rates were highest in the condition where the actor crawled underneath the table towards a target object [all *t*’s(22) > 4.8, *p* < 0.001]. As can also be seen in Fig. 5, accuracy rates in the condition where crawling took place beside the table with no target were also slightly higher than the two remaining conditions [all *t*’s(22) > 3.0, *p* <= 0.006]. This effect was driven by the stimuli of one specific actor, who acted only in two out of ten movies per condition.

The two by two repeated measures ANOVA on the *d’*s, again mirrors the results of the accuracy data, with a main effect of Context [*F*(1,22) = 10.4, *p* = 0.004], a main effect of Target object [*F*(1,22) = 12.1, *p* = 0.002], and a significant interaction [*F*(1,22) = 109, *p* < 0.001]. Post hoc paired-samples *t*-tests were conducted to investigate the two main effects. The Underneath table conditions appeared to have higher *d’*s (*M* = 0.82) than the Beside table conditions [*M* = 0.43; *t*(22) = 3.2, *p* = 0.004]. Furthermore, the Target object conditions yielded more sensitive action predictions (*M* = 0.80) than the No target object conditions [*M* = 0.45, *t*(22) = 3.5, *p* = 0.002]. To study the interaction effect, paired-samples *t*-tests were conducted comparing the four separate conditions. The *d’*s were highest in the condition where crawling took place underneath the table towards a target object (*M* = 1.49), compared to the three other conditions [*t*’s(22) at least equaled 4.2 with all *p*’s < 0.001]. Comparable to the accuracy of results, the Beside table with Target object condition yielded slightly higher *d’*s (*M* = 0.75) than the other two remaining conditions (*M*_Underneath_table × No_target_ = 0.15 and *M*_Beside_table × Target_object_ = 0.12; comparisons between the Beside table × Target object condition and the other two: *t*’s at least equaled 4.4, *p*’s < 0.001). This effect disappeared when excluding the trials of one specific actor. The main and interaction effects then remained significant. The *d’*s of the Beside table with No target condition were then no longer systematically higher than the two remaining conditions [comparison with the condition Beside table with Target object: *t*(22) = 2.3, *p* = 0.03; comparison with the Underneath table with No target object condition: *t*(22) = 1.13, n.s.].

### Conclusion

The results of Experiment 2B are in line with those of Experiment 2A, as action predictions were more accurate when the actor moved to the target object and was constrained in her action by the action context. Given that these predictions were made in the absence of visual information about the position of the target object itself, these findings suggest a crucial role of movement kinematics in action predictions. The *d’* analysis shows the same results as the accuracy data, indicating that this is not just a response bias.

Both the accuracy data and the *d’* analysis show that the trials of one of the actors yielded slightly better predictions in the condition where crawling took place beside the table with no target object compared to the Underneath table with No target and the Beside table with Target object conditions. As mentioned in the “Method” section, this actor was only included in two out of ten trials per condition as she acted in a slightly different way than the other two actors. Apparently, this difference in movements between the actors influenced the prediction accuracy of the observers.

## Discussion

The current study investigated the role of visual information about target objects, situational constraints and movement kinematics for action predictions. The results of Experiment 1 show that observers are more accurate in their predictions of the next move of an actor if the action is object-directed and constrained by the situational context. Experiment 2A and 2B show that these predictions are based on the movement kinematics of the actor. Thus, people act in a more predictable manner if they are moving towards a target object and are constrained by their physical environment. This goal-directedness which resides in the movements of the actor can be effectively detected and used for predictions by the observers.

The present study was the first to test how action prediction is affected by the combination of target object information, situational constraints and movement kinematics. So far, theoretical and computational studies on action prediction suggest that action predictions are based on information about target objects and situational constraints (Gergely & Csibra, 2003; Baker et al., 2009). In Experiment 1, we replicated these findings, and the results clearly show that action prediction accuracy is highest when the action includes a target object and a situational constraint. However, from Experiment 1, it was unclear what the contribution of the actor’s kinematics was to these predictions. Previous work on action observation suggests that action representations are hierarchically organized (Grafton & Hamilton 2007), such that goals are more important than means. Making the kinematics incongruent with the target of the action, as in Experiment 2A, might therefore have led to a similar pattern of action prediction accuracies as in Experiment 1. Yet, the data of Experiment 2A show the reversed pattern of results, indicating a crucial role for movement kinematics in action prediction. The results of Experiment 2B confirm this, as the absence of visual information about the target object still led participants to be more accurate in their predictions of the constrained object-directed actions compared to the other actions. In line with our results, recent empirical work indicates that movement kinematics may affect action predictions (Sartori et al., 2011; Graf et al., 2007; Stadler et al., 2012).

Although typically mentioned in the literature on action perception, the importance of movement kinematics for predicting the actions observed is undervalued. That is, it is often emphasized that actions with similar kinematics can have different goals (Kilner et al., 2007; Jacob & Jeannerod, 2005), and vice versa, similar goals can be achieved with different kinematics. Furthermore, actions with different kinematics but the same goal lead to similar activity in specific mirror neurons in monkeys (Fogassi et al., 2005), which also seems to hold for MNS activity in humans (Gazzola, Rizzolatti, Wicker, & Keysers, 2007). In addition, in behavioral studies, action goals appear to dominate the means to achieve the goal. For instance, imitation studies show that goals are imitated while means are mostly neglected (Bekkering, Wohlschläger, & Gattis, 2000; Wohlschläger & Bekkering, 2002). In reaction time studies, goal-objects evoke stronger interference effects than, for instance, means (van Elk et al., 2008) or spatial information (Bach et al., 2005). Goals seem to be the leading factor in the action hierarchy, whereas movement kinematics are the lowest level in this hierarchy (Grafton & Hamilton, 2007; Hamilton & Grafton, 2007).

However, there are indications that movement kinematics are processed and used by observers. For instance, kinematics of observed actions have been shown to affect automatic imitation, even when the stimulus material is very abstract, such as consisting of a single dot (Bisio, Stucchi, Jacono, Fadiga, & Pozzo, 2010). Furthermore, movement kinematics can form the basis of action predictions, as illustrated by the current study. In a similar vein, other studies have reported that subtle changes in the kinematics of an observed action can be used to predict action targets (Neal & Kilner, 2010). Already in infancy, movement kinematics such as the grip aperture of the actor can form the basis for expectations about which the target object will be grasped (Daum, Vuori, Prinz, & Aschersleben, 2009). Likewise, infants can predict which target will be used based on how a multiple purpose tool is handled (Paulus, Hunnius, & Bekkering, 2011a). This means that the movements of the actor reveal that what the target object will be, before this target has been reached. Another example is that observers can predict whether a basketball shot will be in or out, based on the first few moments of the action (Aglioti, Cesari, Romani, & Urgesi, 2008). Interestingly, professional basketball players need less frames of the same video stimuli to come to an accurate prediction of the outcome and are more accurate than novice players. With experience, people can thus become more sensitive to the subtle differences in the movement patterns.

Taking together our results and the previous findings, the importance of movement kinematics and its role in action prediction becomes somewhat clearer. There are many situations in which the goal of an observed actor is unambiguous. In these cases, kinematics might safely be neglected. However, if the scene shows multiple goal objects or locations, movement characteristics can serve as a cue for predicting what the goal will be. This might for instance be the case when predictions are made about which object a multiple purpose tool will be applied to (Paulus et al. 2011a). Secondly, if we compare actions with similar end locations, but in one case in which a goal will be reached, and in the other case not, kinematics can also play a role in predictions. This holds for instance in a situation in which observers have to judge whether a shot at the goal is in or out (Aglioti et al., 2008), and also for our study in which the one action is object-directed and the other is not.

The stimuli of one actor produced slightly higher prediction accuracy scores than the others in one of the conditions of Experiment 2B. This suggests that there are at least some individual differences in the predictability of actions. This small difference in accuracy is related to one of the actors, and it only emerged in Experiment 2B, while the observed movements were exactly the same as in Experiment 1 and 2A. Apparently, the occlusion of the target object led the participants to direct more attention to the actual movements. This strengthens our case that the obtained results are grounded in the movements of the actors.

The results of the current study show that participants may rely on movement kinematics of an actor when making predictions about the path of the actor. To what extend these results can be generalized to other situations remains to be studied. The actions were observed from a third-person perspective, possibly making it more difficult for observers to predict how they themselves would act in that situation. Studies on MNS activity are still inconclusive about whether first person perspectives give rise to stronger motor involvement or not (Alaerts, Heremans, Swinnen, & Wenderoth, 2009; Keysers et al. 2004; Schaefer, Xu, Flor, & Cohen, 2009). To what extent people vary in the goal-directedness of their movements needs also to be studied more carefully.

A question related to this is: what movement cues do observers use for action predictions? What defines the goal-directedness in the movements of actors? There are several parameters known from action production studies which might affect the predictability of the observed actions. First of all, when approaching an obstacle, velocity is normally reduced and step width is increased already several steps before arriving at the obstacle (Vallis & McFadyen, 2003). In our study, the table functioned as an obstacle in the conditions in which the actor crawled underneath the table. Consequently, her deceleration before switching to crawling might have been stronger when confronted with the table. Second, studies on walking behavior show that larger steps combined with higher speed lead to less predictable steps (Jordan, Challis, & Newell, 2007). Step size and speed may therefore function as a parameter for predictions of observed actions. Furthermore, actions with a wider range of end locations take less time to complete than actions which are tightly constrained (Fitts, 1954), and action perception has been shown to be sensitive to this phenomenon (Grosjean, Shiffrar, & Knoblich, 2007). In the object-directed conditions of our study, the end location was more strongly bound in space than the not-objected directed conditions, which may have influenced the movements of the actors. Other parameters which may influence the predictability of observed actions are head orientation, head movements and arm movements. Pelz, Hayhoe, & Loeber (2001), for instance, show that in a naturalistic task, the pattern of head, eye and hand movements depends on the task context. To what extent action prediction is influenced by all of these movement parameters is still unknown. More experimental research is needed in which each of these factors is carefully manipulated to unravel that which type of movement cues are used in the prediction of observed actions.

In conclusion, our results show that people predict actions based on target objects and situational constraints. Predictions of ongoing actions are more accurate and sensitive if the observed action is constrained by the context and object-directed. For their predictions, observers use subtle movement cues of the observed actor, rather than direct visual information about target objects and context. The action context and target objects thus enhance predictions of an observed ongoing action, through the movement kinematics of the actor.
